# Strain-dependent variation in the early transcriptional response to CNS injury using a cortical explant system

**DOI:** 10.1186/1742-2094-8-122

**Published:** 2011-09-26

**Authors:** David J Graber, Brent T Harris, William F Hickey

**Affiliations:** 1 Dept. Pathology, Dartmouth Medical School, Lebanon, New Hampshire, USA; 2 Dept. Pathology, Georgetown University School of Medicine, Washington D.C., USA; 3 Dept. Neurology, Georgetown University School of Medicine, Washington D.C., USA

**Keywords:** neuroimmunology, cytokine, chemokine, cerebral cortex, CD74, antisecretory factor, explant

## Abstract

**Background:**

While it is clear that inbred strains of mice have variations in immunological responsiveness, the influence of genetic background following tissue damage in the central nervous system is not fully understood. A cortical explant system was employed as a model for injury to determine whether the immediate transcriptional response to tissue resection revealed differences among three mouse strains.

**Methods:**

Immunological mRNAs were measured in cerebral cortex from SJL/J, C57BL/6J, and BALB/cJ mice using real time RT-PCR. Freshly isolated cortical tissue and cortical sections incubated in explant medium were examined. Levels of mRNA, normalized to β-actin, were compared using one way analysis of variance with pooled samples from each mouse strain.

**Results:**

In freshly isolated cerebral cortex, transcript levels of many pro-inflammatory mediators were not significantly different among the strains or too low for comparison. Constitutive, baseline amounts of CD74 and antisecretory factor (ASF) mRNAs, however, were higher in SJL/J and C57BL/6J, respectively. When sections of cortical tissue were incubated in explant medium, increased message for a number of pro-inflammatory cytokines and chemokines occurred within five hours. Message for chemokines, IL-1α, and COX-2 transcripts were higher in C57BL/6J cortical explants relative to SJL/J and BALB/cJ. IL-1β, IL-12/23 p40, and TNF-α were lower in BALB/cJ explants relative to SJL/J and C57BL/6J. Similar to observations in freshly isolated cortex, CD74 mRNA remained higher in SJL/J explants. The ASF mRNA in SJL/J explants, however, was now lower than levels in both C57BL/6J and BALB/cJ explants.

**Conclusions:**

The short-term cortical explant model employed in this study provides a basic approach to evaluate an early transcriptional response to neurological damage, and can identify expression differences in genes that are influenced by genetic background.

## Background

Inbred strains of mice with identical or nearly identical genotypes have been developed and used extensively in experimental research. They provide a valuable means to study genetic influence on various biological determinants. Susceptibility to, or the severity of, experimental models of neurological disease and injury is often strain-dependent [1-3]. In such systems, inflammatory mediators and immunological activation are recognized as key factors. Gene linkage analysis of hybrids from two strains of mice and rats have implicated many immunologically relevant genes that may regulate in clinical susceptibility or severity [2,4-15].

SJL/J mice are a strain commonly used in animal models of neurological disease. Variations in immune responsiveness have also been well defined between C57BL/6J and BALB/cJ mice in non-CNS tissues. It is not fully known whether the immediate response to injury in CNS tissue differs among these strains. A simple ex vivo system was devised to address this. The transcriptional response of inflammatory-related genes was measured in cerebral cortical tissue that was incubated in explant medium for less than five hours from resection. The inflammation-related transcriptional targets selected for analysis were based on their previously documented involvement in models of neurological disease and injury [16-18]. This included pro-inflammatory cytokines, chemokines, CD74, and antisecretory factor. CD74 is differentially regulated among inbred strains following CNS injury [4,19]. Antisecretory factor is an understudied molecule with anti-inflammatory activity that has been implicated in severity of experimental autoimmune encephalomyelitis [20], a model system known to exhibit well established strain-dependent variability [21,22]. In this study, the levels of mRNAs were compared in freshly isolated cerebral cortex and cortical explants among three mouse strains. A classic injury response of pro-inflammatory mediators was observed in cortical explants, yet differences based on genetic background were also observed.

## Methods

### Animals

The Institutional Animal Care and Use Committee at Dartmouth College approved all experimental protocols. All mice were obtained from Jackson Laboratory (Bar Harbor, ME). SJL/J (n = 11), C57BL/6J (7), and BALB/cJ (11) strains were housed at Borwell Animal Facility for several weeks before use in cortical explant experiments. All mice were female with an average age of 3.9 ± 0.6 months. Only female mice were used in this study to avoid gender differences that are well documented in the SJL/J strain, for which a polymorphism on the Y chromosome has been implicated [23].

### Cortical Explants

Mice were euthanized via halothane over-exposure and then decapitated. Brains were removed and set in an acrylic brain matrices (Braintree Scientific, Braintree, MA) where two 1-mm-thick coronal sections positioned within 2 mm from either side of bregma were cut using a razor blade. Cortex was dissected at the corpus callosum and the midline producing four sections per mouse brain. One section of cortex was processed for RNA isolation immediately to determine basal mRNA levels. Other sections of cortical tissue was placed in individual wells of a 48-well Falcon tissue culture plate containing 0.5 ml of pre-warmed DMEM/High Glucose medium (Thermo Scientific HyClone, Rockford, IL ) supplemented with fetal bovine serum (FBS; 10%; Thermo Scientific HyClone), L-glutamine (2 mM), and penicillin (100 units/ml)/streptomycin (100 ug/ml). Explants were placed in a humidified incubator at 37°C with 5% CO_2 _for designated times. The elapsed time from euthanasia to commencement of incubation of cortical explants was less than ten minutes.

### Quantitative real time reverse transcription (RT)-PCR

Cortical explants were stored immediately in RNAlater solution (Invitrogen, Carlsbad, CA) for one day at 4°C and then stored at -80°C until RNA isolation. RNA was extracted using TRIzol Reagent (Invitrogen). Eluted RNA was quantified by spectrophotometry and 1 ug was reverse-transcribed using qScript cDNA SuperMix (Quanta Biosciences, Gaithersburg, MD). Quantitative real-time PCR was performed using PerfeCTa SYBR Green FastMix with low ROX (Quanta Biosciences), 4 ng sample cDNA, and 300 nM of a RT-PCR primer set (IDT, San Jose, CA) listed in Table 1. Settings for analysis using an ABI 7500 machine were as follows: initial denaturation (95°C/3 min) was followed by 50 cycles of denaturation (95°C/15 s) and primer annealing (60°C/45 s). A melt curve was performed on all samples for quality control. The relative quantity of gene expression was analyzed by the 2^(-ΔΔCt) ^method with normalization to the endogenous control β-actin.

**Table 1:** Oligonucleotide primer sets used in quantitative real time RT-PCR analysis

	Sense	Primer Sequence	Amplicon Size	Assession#	Name
β-Actin	Forward	GGCTGTATTCCCCTCCATC	141 bp	NM_007393.2	actin, beta, cytoplasmic
	Reverse	ATGCCATGTTCAATGGGGTA			
ASF	Forward	CAGATCGCCTACGCCATGCAGA	81 bp	NM_008951.1	antisecretory factor
	Reverse	GGCTGAGCTGGCATCCATGTCA			
CCL2	Forward	ACCACCATGCAGGTCCCTGTCAT	75 bp	NM_011333.3	chemokine (C-C motif) ligand 2 (MCP-1)
	Reverse	AGCCAACACGTGGATGCTCCAG			
CCL3	Forward	ACCAGCAGCCTTTGCTCCCA	141 bp	NM_011337.2	chemokine (C-C motif) ligand 3 (MIP-1alpha)
	Reverse	TCCTCGCTGCCTCCAAGACTCT			
CCL4	Forward	TGCTCGTGGCTGCCTTCTGT	99 bp	NM_013652.2	chemokine (C-C motif) ligand 4 (MIP-1beta)
	Reverse	TGTGAAGCTGCCGGGAGGTGTA			
CD74	Forward	CATGGATGACCAACGCGAC	101 bp	NM_010545.3	invariant polypeptide of major histocompatibility complex, class II antigen-associated
	Reverse	TGTACAGAGCTCCACGGCTG			
CiiTA	Forward	GCATGTTGCACACCAGCTCCCT	135 bp	NM_007575.2	major histocompatibility complex class II transactivator
	Reverse	ACGCCAGTCTGACGAAGGTCCA			
COX-2	Forward	CAGACAACATAAACTGCGCCTT	71 bp	NM_011198.3	prostaglandin-endoperoxide synthase 2 (Ptgs2)
	Reverse	GATACACCTCTCCACCAATGACC			
IL-1α	Forward	TACTCGTCGGGAGGAGACGACTCT	107 bp	NM_010554.4	interleukin 1 alpha
	Reverse	TCCTTCAGCAACACGGGCTGGT			
IL-1β	Forward	CCTTCCAGGATGAGGACATGA	71 bp	NM_008361.3	interleukin 1 beta
	Reverse	TGAGTCACAGAGGATGGGCTC			
IL-6	Forward	GAGGATACCACTCCCAACAGACC	141 bp	NM_031168.1	interleukin 6
	Reverse	AAGTGCATCATCGTTGTTCATACA			
IL-12 P35	Forward	GCATGCTGGTGGCCATCGATGA	130 bp	NM_008351.1	interleukin 12, alpha subunit p35
	Reverse	GCGTGAAGCAGGATGCAGAGCT			
IL-12/23 P40	Forward	TGTGCTCGTGGCCTGATCCACT	91 bp	NM_008352.2	interleukin 12,23, beta subunit p40
	Reverse	CGCAGCCCTGATTGAAGAGCTGT			
IL-23 P19	Forward	TATGGCTGTTGCCCTGGGTCACT	118 bp	NM_031252.2	interleukin 23, alpha subunit p19
	Reverse	GCATGTGCGTTCCAGGCTAGCA			
TNF-α	Forward	CAAGGGACAAGGCTGCCCCG	109 bp	NM_013693.2	tumor necrosis factor alpha
	Reverse	GCAGGGGCTCTTGACGGCAG			

## Results

### Baseline levels of immunological mRNA in SJL/J cerebral cortex

Freshly isolated sections of cortical tissue from SJL/J mice were immediately processed for RNA to determine baseline levels of fourteen immunological transcripts. β-actin mRNA tissue levels served as a reference amount. All were lower with messages for chemokines, IL-1α and β, IL-6, and IL-23 less than 0.1% of β-actin levels (Table 2).

**Table 2:** Baseline levels of mRNA in SJL/J cerebral cortex

mRNA	Relative amount (% β-actin)
β-actin	100
ASF	14
COX-2	2
CD74	1
TNF	0.4
IL-12 p35	0.4
CiiTA	0.4
CCL4	0.09
IL-1α	0.08
CCL3	0.06
CCL2	0.02
IL-1β	0.02
IL-6	0.01
IL-23 p19	0.009
IL-12/23 p40	0.002

Freshly isolated cortical tissues were processed for RNA. Pooled cDNA samples from eleven SJL/J mice were measured in replicates of at least three. Amount of each mRNA was expressed as a percentage compared the average β-actin mRNA levels.

### Comparison of constitutive mRNA levels in cerebral cortex from three mouse strains

**Figure 1:**
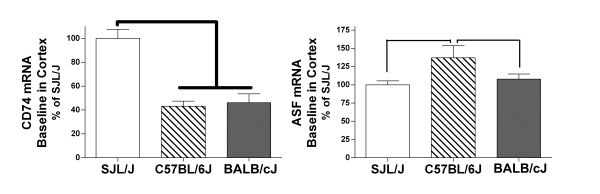
**Comparison of baseline mRNA levels in cortex among mice**. Differential expression of baseline, constitutive CD74 and antisecretory factor (ASF) mRNAs in cerebral cortex among mouse strains. RNA was isolated from resected cortical tissue immediately. β-actin was used as a reference mRNA and values were expressed as percent amount relative to SJL/J cortex + SEM. Pooled cDNA from SJL/J (n = 11), C57BL-6J (7), and BALB/cJ (11) mice were measured in replicates of at least three. Thinner line = P < 0.01 and thicker line = P < 0.001 between strains, Newman-Keuls multiple comparison test.

Levels of immunological transcripts in freshly isolated SJL/J cerebral cortex were compared to baseline levels in freshly isolated C57BL/6J and BALB/cJ cortices. Amounts of CD74 and antisecretory factor (ASF) mRNA differed (Figure 1). CD74 in C57BL/6J and BALB/cJ were lower than 50% of that in SJL/J tissue. ASF was 30% higher in C57BL/6 relative to SJL/J and BALB/cJ tissues. No significant differences in constitutive expression of COX-2 (one-way analysis of variance; *P *= 0.7), TNF-α (0.1), IL-12 p35 (0.08), or CiiTA (0.6) were found. Baseline levels of chemokines, IL-1α and β, IL-6, and IL-23 were negligible (see Table 2) and considered too low to reliably compare among strains based on the detection limits for real-time RT-PCR.

### Immunological transcriptional response in SJL/J cortical explants

**Figure 2:**
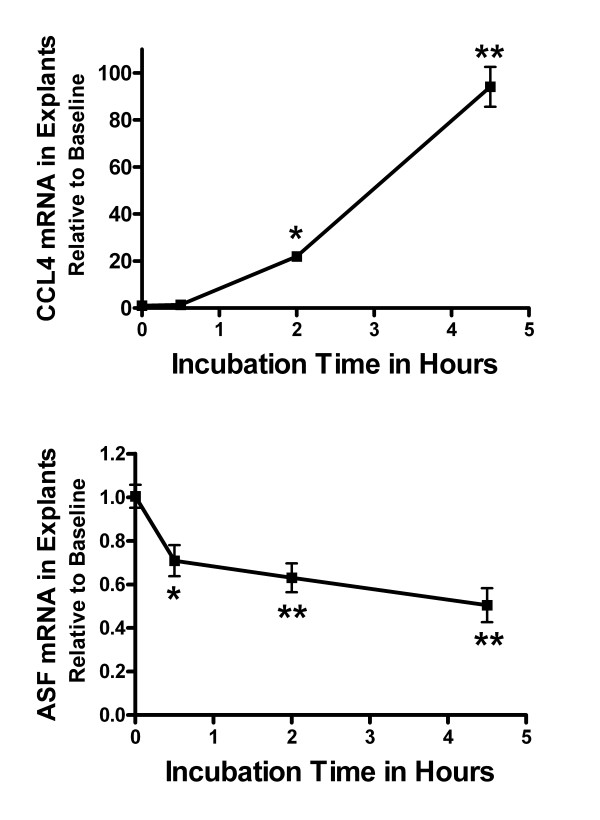
**Change in mRNA expression in cortical explants over time**. Time-dependent change in CCL4 and antisecretory factor (ASF) mRNA expression in SJL/J cortical explants. RNA was isolated from resected cortical tissue after incubation in explant medium for 0, 0.5, 2, and 4.5 hours. Transcript levels were referenced to β-actin mRNA levels. Values were expressed as fold difference relative to freshly isolated cortex (baseline) + SEM. Pooled cDNA from SJL/J mice (n = 4) were measured in replicates of four. * = P < 0.05 and ** = P < 0.01, relative to 0 hours, Dunnett's multiple comparison test.

Tissue levels of mRNA in sections of cortical tissue from SJL/J mice incubated in explant medium were expressed as a fold difference relative to baseline levels in freshly isolated SJL/J cerebral cortex. The quantity of eleven transcripts changed within five hours of incubation (Table 3). Chemokines and pro-inflammatory cytokines increased in cortical explants with the exception to the p35 subunit of IL-12. Message for ASF decreased in a time-dependent manner (Figure 2).

**Table 3:** Change in mRNA levels in SJL/J cortical tissue after incubation in explant medium

mRNA	Fold difference in explants at 4.5 hrs (relative to freshly isolated cortex)	Significance (unpaired t test)
CCL4	912	*P *< 0.001
CCL3	518	*P *< 0.001
IL-12/23 p40	159	*P *< 0.001
IL-1β	98	*P *< 0.0001
IL-6	55	*P *< 0.0001
IL-1α	50	*P *< 0.001
CCL2	15	*P *< 0.01
TNF-α	13	*P *< 0.001
COX-2	3.9	*P *< 0.0001
IL-23 p19	3.3	*P *< 0.01
IL-12 p35	1.1	NS
CD74	0.90	NS
CiiTA	0.61	NS
ASF	0.58	*P *< 0.001

Freshly isolated cortical tissue and cortical tissue incubated in explant medium for 4.5 hours were processed for RNA. Transcript levels were referenced to β-actin mRNA levels. Values were expressed as fold difference in explants relative to freshly isolated cortex. Pooled cDNA from eleven SJL/J mice were measured in replicates of at least three. NS, not significant.

### Comparison of mRNA levels in cortical explant from three mouse strains

**Figure 3:**
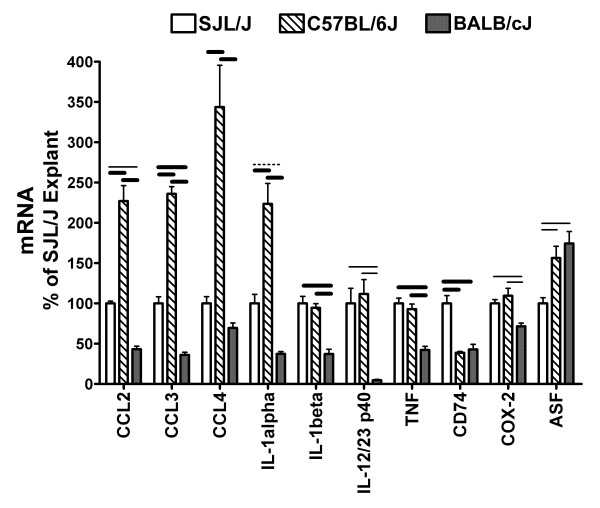
**Comparison of mRNA levels in cortical explant among mice**. Differential expression of immunological mRNAs in cortical explants among mouse strains. RNA was isolated from resected cortical tissue after incubation in explant medium for 4.5 hours. β-actin was used as a reference mRNA and values were expressed as percent amount relative to SJL/J cortical explants + SEM. Pooled cDNA from SJL/J (n = 11), C57BL/6J (7), and BALB/cJ (11) mice were measured in replicates of at least four. Dotted line = P < 0.05, thinner line = P < 0.01, and thicker line = P < 0.001 between strains, Newman-Keuls multiple comparison test.

**Figure 4:**
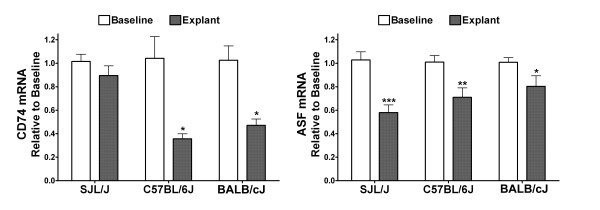
**Change in mRNA expression in cortical explants among mice**. Change in CD74 and antisecretory factor (ASF) mRNA in cortical explants in mouse strains. RNA was isolated from freshly resected cortical tissue (baseline) and cortical explants after incubation for 4.5 hours. Transcript levels were referenced to β-actin mRNA levels. Values were expressed as fold difference relative to baseline + SEM. Pooled cDNA from SJL/J (n = 11), C57BL/6J (7), and BALB/cJ (11) mice were measured in replicates of at least three. * = P < 0.05 and ** = P < 0.01, relative to baseline, unpaired t test.

Sections of cortical tissue from SJL/J, C57BL/6J, and BALB/cJ mice were incubated in explant medium for 4.5 hours. Immunological transcripts in cortical explants from these strains were determined and expressed as a percent of the amount in SJL/J - i.e., for this interstrain comparison the transcript amount for the moieties studied were calculated using the levels found in SJL/J explants as the standard. Transcripts for many pro-inflammatory mediators and antisecretory factor revealed differential tissue levels among strains (Figure 3). C57BL/6J explants had higher mRNA amounts for chemokines and IL-1α relative SJL/J and BALB/cJ explants. IL-1β, TNF-α, IL-12/23 p40, and COX-2 revealed a similar profile with SJL/J and C57BL/6J having similar amounts that were higher than in BALB/cJ explants. No significant difference in abundance of IL-6 (one-way analysis of variance; *P *= 0.6), IL-12 p35 (0.07), IL-23 p19 (0.4), CiiTA (0.1) mRNA were observed among these strains. Since strain differences in CD74 and ASF mRNA were found in freshly isolated cortex and in cortical explants, fold differences within each strain was evaluated. CD74 mRNA was down-regulated in C57BL/6J and BALB/cJ, but not in SJL/J explants relative to baseline levels, while ASF was down-regulated by varying degrees in all three strains (Figure 4).

## Discussion

The data reported in this study establish that differences in the immediate gene response to damage of central nervous system (CNS) tissue occur among mouse strains. Message for several genes involved in CNS injury and neurological diseases with autoimmunity or chronic innate immune activation were strain-dependently altered. Short-term explants of cortical sections provided a reliable system for defining immunologically relevant transcriptional changes in CNS tissue and the model may serve as a cost-effective method to test novel immunomodulating pharmaceuticals.

One millimeter-thick explants of adult cerebral cortex were used in this study. Acute CNS explants of this thickness have been previously demonstrated to induce pro-inflammatory mRNA expression consistent to the temporal profile observed following injury *in vivo *[24,25]. The production of immunologically relevant mRNAs is likely caused by a combination of tissue damage at the periphery of the explant due to tissue sectioning and axotomy of projection neurons throughout the explant, and some undefined amount of ischemia in the center of the explant. Pro-inflammatory mRNA increase occurs in cortex within hours following lesioning [26] or ischemia [27,28]*in vivo*. Since there were no established foci of inflammation in the tissue prior to sectioning in our explant model, the influence of the miniscule number of leukocytes in the vasculature would contribute only marginally and is limited to the residual cells in the vasculature at the time of sectioning. Therefore, the extent of the measured pro-inflammatory response is predominantly by resident CNS cells.

Pro-inflammatory cytokines and chemokines expression are activated in cells of the monocyte/macrophage lineage in the innate response to injury or infections. Microglia are considered the primary cell type within the CNS parenchyma that carry out this function. Pattern recognition receptors recognize specific molecules released from damaged host cells or foreign microbes leading to the activation of transcription factors that induce transcription of certain inflammatory genes. Sections of cerebral cortex incubated ex vivo in explant medium were demonstrated to increase mRNA for pro-inflammatory cytokines and chemokines within five hours. The constitutive amounts in uninjured cortical tissue were mostly very low and transcripts that could be confidently quantified did not differ significantly among mouse strains. However the amount measured in cortical explants from these strains was different for several of the transcripts suggesting strain-related alterations in their induction. The chemokines evaluated were CCL2 (MCP-1), CCL3 (MIP-1α), and CCL4 (MIP-1β). These transcripts were found in increasing abundance in BALB/cJ, SJL/J, and C57BL/6J, respectively. Interestingly, the abundance of IL-1α and COX-2 mRNA revealed a similar pattern to the chemokines suggesting these inflammatory mediators could have a common regulatory mechanism that is distinct among these mouse strains. Messages for IL-1β and TNF-α were similar in SJL/J and C57BL/6 explants, but higher than BALB/cJ. Taken together, BALB/cJ appears to have a dampened immunological response to tissue damage in cerebral cortex.

Expression of genes associated with autoimmunity was also examined in this study. Experimental autoimmune encephalomyelitis is a widely studied autoimmune model, and involves infiltration of CD4+ lymphocytes and immunological activation of microglia within the CNS [21,22,29]. Although MHC class II haplotype and its binding to specific myelin autoantigen play a pivotal role in this model, non-MHC class II effectors are also implicated [30]. BALB/cJ is EAE resistant while SJL/J and C57BL/6J are susceptible [2,3,31]. Cytokines IL-12 and IL-23 are implicated in the pathogenesis of autoimmune diseases including EAE [17]. The p35 subunit of IL-12 and p19 subunit of IL-23 form respective cytokines with a common p40 subunit. Blocking the IL-12/23 p40 subunit with inhibiting antibodies is effective in non-CNS autoimmune diseases such as psoriasis [32]. In cortical explants, the p40 mRNA was upregulated. Its levels were considerably lower in the resistant BALB/cJ explants. This suggests that inherent difference within the CNS tissues may contribute to strain susceptibility to autoimmunity.

Antisecretory factor (ASF) has been shown to affect the severity of EAE. Blocking its activity with an inhibiting antibody increases clinical severity implying it has an anti-inflammatory property [20]. Our results showed that differences in ASF mRNA expression occurred in normal cortical tissue and in cultured cortical explants. C57BL/6J mice had high constitutive ASF mRNA. Its levels decreased after injury in cortical explants in each strain, but to a lesser degree in BALB/cJ. This supports the hypothesis that higher amounts of ASF due to genetic background may contribute to EAE resistance.

Antigen presentation by MHC class II is critical for many autoimmune diseases. CD74 (invariant chain, Ii) acts as an MHC class II chaperone [33,34]. CD74 mRNA was higher in SJL/J cortex relative to BALB/cJ and C57BL/6. A similar trend among these strains was reported in facial nucleus two weeks following facial nerve axotomy [19]. Our study revealed that higher levels were found in uninjured cortex and that these differences were increased further in explants due to down-regulation in C57BL/6J and BALB/cJ tissues. CD74 also mediates transcription by NF-κB [35,36] and regulates dendritic cell migration [37]. This suggests its altered expression among strains could influence a wide range of effects.

Now, identifying the inherent genetic polymorphisms that control the variations in transcriptional response to an injury stimulus among strains is important for understanding genetically variable responses to a spectrum of neurological disorders. Approaches such as quantitative trait locus analysis and/or haplotype-based computational genetic mapping can be utilized with the cortical explant model. Haplotype-based computational genetic mapping has recently pinpointed genetic variation of Nalp1 as a contributor to interstrain differences in the inflammatory response to injured skin [38]. It is important to recognize that the involvement of multiple genes may be required and that such analyses will likely require data from additional strains and transcripts.

## Conclusions

The genes expressed differentially in cortical explants derived from disparate strains of mice reveal that genetic background can influence immediate response to neurological damage within the CNS. The straightforward approach described in this study may help uncover the inherent regulatory mechanism that control altered immunological responsiveness and perhaps neurological disease susceptibility in future studies.

## List of abbreviations

ASF: antisecretory factor; CCL: CC chemokine ligand; CiiTA: class II transactivator; COX: cyclooxygenase; CNS: central nervous system; FBS: fetal bovine serum; IL: interleukin; RT-PCR: reverse transcription- polymerase chain reaction; TNF: tumor necrosis factor

## Competing interests

The authors declare that they have no competing interests.

## Authors' contributions

DJG carried out the experiments and evaluated the data. DJG, BTH, and WFH participated in the design and assisted with the preparation of the manuscript. All authors have read and approved the final version of the manuscript.

## References

[B1] Heiman-Patterson TD Deitch JS Blankenhorn EP Erwin KL Perreault MJ Alexander BK Byers N Toman I Alexander GM Background and gender effects on survival in the TgN(SOD1-G93A)1Gur mouse model of ALS J Neurol Sci 2005 236 1 7 10.1016/j.jns.2005.02.006 16024047

[B2] Encinas JA Lees MB Sobel RA Symonowicz C Greer JM Shovlin CL Weiner HL Seidman CE Seidman JG Kuchroo VK Genetic analysis of susceptibility to experimental autoimmune encephalomyelitis in a cross between SJL/J and B10. S mice J Immunol 1996 157 2186 92 8757345

[B3] Royle SJ Collins FC Rupniak HT Barnes JC Anderson R Behavioural analysis and susceptibility to CNS injury of four inbred strains of mice Brain Res 1999 816 337 49 10.1016/S0006-8993(98)01122-6 9878817

[B4] Diez M Abdelmagid N Harnesk K Ström M Lidman O Swanberg M Lindblom R Al-Nimer F Jagodic M Olsson T Identification of gene regions regulating inflammatory microglial response in the rat CNS after nerve injury J Neuroimmunol 2009 212 82 92 10.1016/j.jneuroim.2009.05.004 19525015

[B5] Piehl F Swanberg M Lidman O The axon reaction: Identifying the genes that make a difference Physiol Behav 2007 92 67 74 10.1016/j.physbeh.2007.05.030 17561176

[B6] Swanberg M Harnesk K Ström M Diez M Lidman O Piehl F Linden R Fine mapping of gene regions regulating neurodegeneration PLoS One 2009 4 689 700 10.1371/journal.pone.0005906 PMC2691596 19526058

[B7] Butterfield RJ Sudweeks JD Blankenhorn EP Korngold R Marini JC Todd JA Roper RJ Teuscher C New genetic loci that control susceptibility and symptoms of experimental allergic encephalomyelitis in inbred mice J Immunol 1998 161 1860 7 9712054

[B8] Baker D Rosenwasser OA O'Neill JK Turk JL Genetic analysis of experimental allergic encephalomyelitis in mice J Immunol 1995 155 4046 51 7561115

[B9] Sundvall M Jirholt J Yang HT Jansson L Engström Å Pettersson U Holmdahl R Identification of murine loci associated with susceptibility to chronic experimental autoimmune encephalomyelitis Nat Genet 1995 10 313 7 10.1038/ng0795-313 7545492

[B10] Croxford JL O'Neill JK Baker D Polygenic control of experimental allergic encephalomyelitis in Biozzi ABH and BALB/c mice J Neuroimmunol 1997 74 205 11 10.1016/S0165-5728(96)00219-6 9119975

[B11] Teuscher C Butterfield RJ Ma RZ Zachary JF Doerge RW Blankenhorn EP Sequence polymorphisms in the chemokines Scya1 (TCA-3), Scya2 (monocyte chemoattractant protein (MCP)-1), and Scya12 (MCP-5) are candidates for eae7, a locus controlling susceptibility to monophasic remitting/nonrelapsing experimental allergic encephalomyelitis J Immunol 1999 163 2262 6 10438970

[B12] Marta M Stridh P Becanovic K Gillett A Öckinger J Lorentzen JC Jagodic M Olsson T Multiple loci comprising immune-related genes regulate experimental neuroinflammation Genes Immun 2009 11 21 36 19675581 10.1038/gene.2009.62

[B13] Teuscher C Doerge RW Fillmore PD Blankenhorn EP eae36, a locus on mouse chromosome 4, controls susceptibility to experimental allergic encephalomyelitis in older mice and mice immunized in the winter Genetics 2006 172 1147 53 16299394 10.1534/genetics.105.049049 PMC1456213

[B14] Butterfield RJ Blankenhorn EP Roper RJ Zachary JF Doerge RW Teuscher C Identification of genetic loci controlling the characteristics and severity of brain and spinal cord lesions in experimental allergic encephalomyelitis Am J Pathol 2000 157 637 45 10.1016/S0002-9440(10)64574-9 10934166 PMC1850129

[B15] Spach KM Case LK Noubade R Petersen CB McElvany B Zalik N Hickey WF Blankenhorn EP Teuscher C Multiple linked quantitative trait loci within the Tmevd2/Eae3 interval control the severity of experimental allergic encephalomyelitis in DBA/2J mice Genes Immun 2010 11 649 59 10.1038/gene.2010.40 20861860 PMC2995842

[B16] Wang CX Shuaib A Involvement of inflammatory cytokines in central nervous system injury Prog Neurobiol 2002 67 161 72 10.1016/S0301-0082(02)00010-2 12126659

[B17] Kroenke MA Carlson TJ Andjelkovic AV Segal BM IL-12-and IL-23-modulated T cells induce distinct types of EAE based on histology, CNS chemokine profile, and response to cytokine inhibition J Exp Med 2008 205 1535 41 10.1084/jem.20080159 18573909 PMC2442630

[B18] Fischer FR Santambrogio L Luo Y Berman MA Hancock WW Dorf ME Modulation of experimental autoimmune encephalomyelitis: effect of altered peptide ligand on chemokine and chemokine receptor expression J Neuroimmunol 2000 110 195 208 10.1016/S0165-5728(00)00351-9 11024550

[B19] Harnesk K Swanberg M Diez M Olsson T Piehl F Lidman O Differential nerve injury-induced expression of MHC class II in the mouse correlates to genetic variability in the type I promoter of C2ta J Neuroimmunol 2009 212 44 52 10.1016/j.jneuroim.2009.04.019 19481818

[B20] Davidson TS Hickey WF Antisecretory factor expression is regulated by inflammatory mediators and influences the severity of experimental autoimmune encephalomyelitis J Leukoc Biol 2004 76 835 44 10.1189/jlb.0204085 15277566

[B21] Furlan R Cuomo C Martino G Animal models of multiple sclerosis Methods Mol Biol 2009 549 157 73 10.1007/978-1-60327-931-4_11 19378202

[B22] Gold R Linington C Lassmann H Understanding pathogenesis and therapy of multiple sclerosis via animal models: 70 years of merits and culprits in experimental autoimmune encephalomyelitis research Brain 2006 129 1953 71 10.1093/brain/awl075 16632554

[B23] Spach KM Blake M Bunn JY McElvany B Noubade R Blankenhorn EP Teuscher C Cutting edge: the Y chromosome controls the age-dependent experimental allergic encephalomyelitis sexual dimorphism in SJL/J mice J Immunol 2009 182 1789 93 10.4049/jimmunol.0803200 19201829 PMC2658649

[B24] Pan JZ Ni L Sodhi A Aguanno A Young W Hart RP Cytokine activity contributes to induction of inflammatory cytokine mRNAs in spinal cord following contusion J Neurosci Res 2002 68 10.1002/jnr.10215 12111861

[B25] Rice T Larsen J Rivest S Yong VW Characterization of the early neuroinflammation after spinal cord injury in mice J Neuropathol Exp Neurol 2007 66 184 10.1097/01.jnen.0000248552.07338.7f 17356380

[B26] Rostworowski M Balasingam V Chabot S Owens T Yong VW Astrogliosis in the neonatal and adult murine brain post-trauma: elevation of inflammatory cytokines and the lack of requirement for endogenous interferon-γ J Neurosci 1997 17 3664 9133389 10.1523/JNEUROSCI.17-10-03664.1997 PMC6573669

[B27] Wang X Yue TL Barone FC White RF Gagnon RC Feuerstein GZ Concomitant cortical expression of TNF-α and IL-1β mRNAs follows early response gene expression in transient focal ischemia Mol Chem Neuropathol 1994 23 103 14 10.1007/BF02815404 7702701

[B28] Berti R Williams AJ Moffett JR Hale SL Velarde LC Elliott PJ Yao C Dave JR Tortella FC Quantitative real-time RT-PCR analysis of inflammatory gene expression associated with ischemia-reperfusion brain injury J Cereb Blood Flow Metab 2002 22 1068 79 12218412 10.1097/00004647-200209000-00004

[B29] Murphy ÁC Lalor SJ Lynch MA Mills KHG Infiltration of Th1 and Th17 cells and activation of microglia in the CNS during the course of experimental autoimmune encephalomyelitis Brain Behav Immun 2010 24 641 51 10.1016/j.bbi.2010.01.014 20138983

[B30] Happ M Wettstein P Dietzschold B Heber-Katz E Genetic control of the development of experimental allergic encephalomyelitis in rats. Separation of MHC and non-MHC gene effects J Immunol 1988 141 1489 94 2457618

[B31] Teuscher C Hickey WF Grafer CM Tung KSK A common immunoregulatory locus controls susceptibility to actively induced experimental allergic encephalomyelitis and experimental allergic orchitis in BALB/c mice J Immunol 1998 160 2751 9 9510176

[B32] Leonardi CL Kimball AB Papp KA Yeilding N Guzzo C Wang Y Li S Dooley LT Gordon KB Efficacy and safety of ustekinumab, a human interleukin-12/23 monoclonal antibody, in patients with psoriasis: 76-week results from a randomised, double-blind, placebo-controlled trial (PHOENIX 1) The Lancet 2008 371 1665 74 10.1016/S0140-6736(08)60725-4 18486739

[B33] Roche PA Teletski CL Stang E Bakke O Long EO Cell surface HLA-DR-invariant chain complexes are targeted to endosomes by rapid internalization Proc Natl Acad Sci USA 1993 90 8581 5 10.1073/pnas.90.18.8581 8397411 PMC47401

[B34] Cresswell P Assembly, transport, and function of MHC class II molecules Annu Rev Immunol 1994 12 259 91 10.1146/annurev.iy.12.040194.001355 8011283

[B35] Teo BHD Wong SH MHC class II-associated invariant chain (Ii) modulates dendritic cells-derived microvesicles (DCMV)-mediated activation of microglia Biochem Biophys Res Commun 2010 400 673 8 10.1016/j.bbrc.2010.08.126 20816669

[B36] Matza D Kerem A Medvedovsky H Lantner F Shachar I Invariant chain-induced B cell differentiation requires intramembrane proteolytic release of the cytosolic domain Immunity 2002 17 549 60 10.1016/S1074-7613(02)00455-7 12433362

[B37] Faure-Andre G Vargas P Yuseff MI Heuze M Diaz J Lankar D Steri V Manry J Hugues S Vascotto F Regulation of dendritic cell migration by CD74, the MHC class II-associated invariant chain Science 2008 322 1705 10 10.1126/science.1159894 19074353

[B38] Hu Y Liang D Li X Liu HH Zhang X Zheng M Dill D Shi X Qiao Y Yeomans D Carvalho B Angst MS Clark JD Peltz G The Role of Interleukin-1 in Wound Biology. Part I: Murine In Silico and In Vitro Experimental Analysis Anesth Analg 2010 111 1525 33 10.1213/ANE.0b013e3181f5ef5a 20889942

